# Granzyme B knockdown suppresses cell proliferation, migration and invasion of fibroblast-like synoviocytes in rheumatoid arthritis

**DOI:** 10.3389/fcell.2026.1679672

**Published:** 2026-06-17

**Authors:** Wei Zhang, Weike Tian, Chao Zhang

**Affiliations:** Department of Orthopaedics, Affiliated Hospital of North Sichuan Medical College, Nanchong, China

**Keywords:** AKT, fibroblast-like synoviocytes, granzyme B, MH7A, PI3K, rheumatoid arthritis

## Abstract

**Background:**

Rheumatoid arthritis (RA) is a chronic autoimmune disease characterized by progressive synovial inflammation, cartilage and bone destruction. Although granzyme B (GZMB) can prevent osteocartilaginous destruction and promote bone formation, its effects and molecular mechanisms in RA remain unclear. We therefore explored the effects of GZMB on RA development at the cellular level.

**Methods:**

We analyzed the effects of GZMB on MH7A cell proliferation using Cell Counting Kit-8 assays. Migration and invasion were detected using wound healing and Transwell® assays, respectively, and the cell cycle and apoptosis were assessed using flow cytometry. Additionally, 24 Sprague-Dawley (SD) rats were divided into sham, RA, and RA + GZMB inhibitor groups. Pathological changes, inflammatory factors, and related proteins were assessed in each group after treatment. Furthermore, we evaluated correlations between GZMB and the phosphoinositide 3-kinase (PI3K)/protein kinase B (AKT) pathway.

**Results:**

Granzyme B suppressed cell proliferation, migration and invasion *in vitro*, promoted apoptosis, and increased the proportion of cells in the S and G2 phases. Additionally, histological staining revealed that after GZMB inhibitor treatment, the articular cartilage surface of rats in the GZMB inhibitor group was smooth, and synovial tissue hyperplasia and inflammatory cell infiltration were reduced. Moreover, GZMB regulated expression of the PI3K/AKT signaling pathway during RA treatment *in vitro* and *in vivo*.

**Conclusion:**

Our findings suggest that GZMB knockdown suppresses cell proliferation, migration and invasion of fibroblast-like synoviocytes in rheumatoid arthritis, and alleviates the progression of RA rats. This therapeutic mechanism may involve regulation of the PI3K/AKT pathway.

## Introduction

Rheumatoid arthritis (RA) is a chronic autoimmune disease (Radu and Bungau, 2021), and easily causes teratogenesis and disability, which seriously affect the quality of life of patients (Smolen et al., 2016). The main clinical characteristics of RA are chronic synovial inflammation and aggressive destruction of joints that manifest as progressive and symmetrical multi-joint chronic inflammation (Grav et al., 2024). In the early stage of RA patients, the deformity and destruction of the hand and wrist joints become evident. Subsequently, swelling, pain, deformity, and immobility spread to the patient’s knees, buttocks and other major joints during the middle and later stages of the disease (Cush, 2022). The pathogenesis of RA is characterized by synovial cell proliferation, thickened synovial lining, inflammatory cell infiltration, pannus formation, and the destruction of soft bone and bone tissue (Lin et al., 2020). The disease course of RA is long, and many affected organs and joints lead to high disability rates (Díaz-González and Hernández-Hernández, 2023). Therefore, finding appropriate treatment strategies is particularly important.

Abnormal migration and invasive fibroblast-like synoviocytes (FLS) are important causes of synovial proliferation and invasion in patients with RA (McInnes and Schett, 2011). Fibroblast-like synoviocytes in the synovial lining are transformed under inflammatory stimulation in patients with RA. The invasive capacity, proliferation, and migration of transformed FLS are significantly enhanced, and secreted pro-inflammatory factor and chemokines aggravate bone and cartilage erosion and eventually lead to irreversible joint breakage (Kim et al., 2023). Therefore, suppression of these pathological FLS behaviors has emerged as a potential therapeutic approach in RA. In RA, fibroblast-like synoviocytes become abnormally activated by inflammatory signals, increasing proliferation, migration, and invasive behavior. Fibroblast-like synoviocytes also secrete several pro-inflammatory cytokines and chemokines, which aggravate the pathological process of RA (Song and Lin, 2017). Inhibiting excessive RA-FLS proliferation and promoting apoptosis has been proposed as a potential therapeutic strategy for RA.

Granzyme B (GZMB) is a serine protease best known for its intracellular role in inducing apoptosis of virus-infected or tumor cells via the granule exocytosis pathway of cytotoxic T lymphocytes (CTLs) and natural killer (NK) cells (Zhang et al., 2019; Li et al., 2024). In the context of RA, however, the role of GZMB is complex and remains incompletely understood. Protective roles for GZMB have been proposed. By inducing apoptosis of chronically activated synovial fibroblasts or inflammatory cells, GZMB could theoretically limit synovial hyperplasia and resolve joint inflammation (Bao et al., 2018). Some studies suggest that GZMB can prevent osteocartilaginous destruction and promote bone formation, potentially through cleavage of specific matrix proteins or regulation of osteoclast activity (Collora et al., 2022). However, pathogenic roles for GZMB have also emerged. When released extracellularly, either from degranulating lymphocytes or from activated fibroblast-like synoviocytes (FLS) themselves, GZMB can degrade extracellular matrix components (including aggrecan, fibronectin, and type IV collagen) and process pro-inflammatory cytokines such as IL-1α (Zhang et al., 2023). Elevated GZMB expression has been detected in the synovial tissue and fluid of RA patients, and its levels correlate with disease activity (Zhang et al., 2023). Although GZMB is traditionally recognized for its cytotoxic role in antiviral defense, its proteolytic activity may also degrade extracellular matrix components and promote synovial inflammation and joint destruction in RA. So far, the molecular mechanism by which the expression changes of GZMB are involved in the progression of RA remains unclear.

The PI3K/AKT signaling pathway is closely associated with inflammation (Glaviano et al., 2023). Affected chondrocytes secrete inflammatory transmitters as osteoarthritis develops. This causes excessive activation of the PI3K/AKT signaling pathway, resulting in increased synthesis and release of inflammatory cytokines that promote inflammatory cell migration and invasiveness (Sun et al., 2020). Abnormal activation of the PI3K/AKT/mTOR signaling pathway can promote chondrocyte apoptosis and inhibit autophagy, thus promoting the occurrence and development of osteoarthritis (Lu et al., 2022). Activating the PI3K/AKT signaling pathway causes joint synovial inflammation and bone and cartilage erosion by promoting the tumor-like biological behavior of FLS in RA (Ba et al., 2021). Therefore, blocking PI3K/AKT pathway should alleviate RA development.

Based on the above, the net effect of GZMB in RA may depend on its cellular source, subcellular localization, and the disease stage. Therefore, the present study focuses on FLS-derived GZMB. We examined whether GZMB downregulation reduces MH7A migration/invasion and mitigates RA progression, focusing on PI3K/AKT involvement. Furthermore, by establishing a rat model of RA, we detected *in vivo* whether downregulation of GZMB alleviated the injury in RA rats and whether it regulated the PI3K/AKT pathway.

## Materials and methods

### Cell culture and transfection

We obtained rheumatic fibroblast-like synoviocytes cells (MH7A) (Cell Bank of the Chinese Academy of Sciences (Shanghai, China) that were identified using short tandem repeat (STR) profiling. We assessed *mycoplasma* contamination in all cells using LookOut® *Mycoplasma* PCR Detection Kits (Merck KGaA, Darmstadt, Germany). The cells were incubated at 37 °C under a 5% CO_2_ atmosphere in Roswell Park Memorial Institute (RPMI)-1640 medium containing 10% fetal bovine serum (Gibco, Rockville, MD, USA) and sub-cultured every 2–3 days. The cells were incubated with serum-free medium (500 μL) containing (10 μL each) of Lipofectamine 2000 and small interfering (si)-GZMB or Lipofectamine 2000 and si-NC controls (10 μL each; controls). Additional, theGZMB inhibitor PI-9 were purchased from Boster Biological Technology (A04734, Pleasanton, CA, USA). The MH7A cells were seeded in 24-well plates at 5 × 10^4^ cells/well and cultured overnight, and then the PI-9 (catalog #A04734, Boster Biological Technology, Pleasanton, CA, USA) was added to the culture medium at a final concentration of 50 ng/mL for 48 h. PI-9 was used to validate that the effects observed with si-GZMB knockdown were indeed due to loss of GZMB enzymatic activity rather than off-target effects of siRNA.

### Establishment of animal model

The rat model of RA was established according to a previously reported protocol (Yan et al., 2024). Twenty-four male Sprague–Dawley (SD) rats (250 ± 20 g) were purchased from Shanghai Slack Laboratory Animal Co., Ltd. (Shanghai, China). All rats were housed in standard cages with a light/dark cycle of 12 h at a temperature of 18 °C–25 °C and relative humidity of 65%–70%. The SD rats were randomly divided into three groups using a computer-generated random number sequence: healthy control (sham, n = 6), RA model control (RA, n = 6), and GZMB inhibitor (Calbiochem-Novabiochem, La Jolla, CA, USA) treatment (50 μg/kg, n = 6) groups. All rats except for those in the sham group were injected with 0.3 mL type II collagen emulsion on the tail root, back, and foot pad on the 1st and 8th days of the experiment to establish the RA model. After the model was successfully established, rats in the treatment group were treated with a GZMB inhibitor (Calbiochem-Novabiochem, La Jolla, CA, USA) at 50 μg/kg/day via intraperitoneal injection once daily for 4 weeks. Rats in the sham and RA groups received an equal volume of normal saline via the same route and scheduleonce. Animals were monitored daily. Humane endpoints included: (1) body weight loss ≥20% from baseline, (2) arthritis score ≥8, or (3) inability to reach food or water. Any animal reaching these endpoints was euthanized immediately. No animals reached humane endpoints before the scheduled completion of the study. At the end of the treatment period, all rats were euthanized by sodium pentobarbital overdose (150 mg/kg, intraperitoneal injection). Synovial tissue and serum were collected for further analysis. All experimental and animal care procedures were approved by Affiliated Hospital of North Sichuan Medical College (approval no. 2023-1) and conformed to the Guide for the Care and Use of Laboratory Animals produced by the National Institutes of Health.

### Arthritis scoring

The evaluators responsible for arthritis scoring and histological evaluation was blinded to group allocation. During the treatment process, from day 0 to day 28, the arthritis score was evaluated every week to observe the signs of arthritis. Based on previous studies (Pritzker et al., 2006), the severity of arthritis was scored on a scale of 0–4: 0 for no redness or swelling; 1 for redness and swelling in the toe joints; 2 indicated redness and swelling in both the toe and ankle joints; 3 for redness and swelling below the ankle joint in the foot; 4 for swelling and redness in all joints (including the ankle joint), and the total score for both hind feet was 8.

### Hematoxylin and eosin staining

The synovial tissue of joints was fixed with 4% paraformaldehyde, dehydrated with alcohol, rendered transparent with xylene, embedded in paraffin, and stone wax sections (5 μm) were prepared. Sections were stained with hematoxylin for approximately 30 min, stained with eosin staining solution for 1 min, rinsed, dehydrated, sealed with transparent neutral gum, and observed under an optical microscope (Leica DM2500M; Leica Microsystems, Wetzlar, Germany). Histological evaluation was scored using the Osteoarthritis Research Society International (OARSI) histopathology grading system (Pritzker et al., 2006) in a blinded manner.

### Cell Counting Kit-8 (CCK-8) assays

MH7A cells were seeded (5 × 10^3^/100 μL) into 96-well plates and incubated for 0, 24, 48, and 72 h after treated with si-GZMB, si-NC or PI-9. Thereafter, the cells were incubated for 2 h in 10% CCK-8 solution. Absorbance (OD) was measured at 450 nm using a microplate reader (BioTek, Winooski, VT, USA).

### Wound-healing assay

We seeded cells (5 × 10^4^) into six-well plates cells and incubated them with GZMB (1, 5, 10, 20, or 30 µM), until they reached confluence. Monolayers were scratched with the tips of straws, then non-adherent cells were removed. The cells were incubated in RPMI-1640 medium without fetal bovine serum at 37 °C under a 5% CO_2_ atmosphere for 24 h. The cells were photographed at 0 and 24 h using a light microscope, and cell migration was calculated.

### Transwell® assays

Cell suspensions (100 μL) were added to the upper chambers of Transwell® plates containing solidified diluted Matrigel (100 μL) and 500 μL of medium containing serum was added to the lower chambers and left for 24 h. The cells were stained with 1% crystal violet for 30 min, then the upper layer of cells that did not penetrate the basement membrane were removed using a cotton swab. Invaded cells were visualized using a microscope.

### Flow cytometry

Apoptosis was assessed 48 h after transfection (si-GZMB or si-NC) or after PI-9 treatment. Cells were harvested, washed twice with cold PBS, and resuspended in 1× binding buffer. Cells were then stained with 5 μL of FITC Annexin V and 5 μL of propidium iodide (PI) using the FITC Annexin V Apoptosis Detection Kit (BD Biosciences, San Jose, CA, USA) according to the manufacturer’s instructions. After incubation for 15 min at room temperature in the dark, samples were analyzed within 1 h using a BD FACSCanto II flow cytometer (BD Biosciences). For each sample, 10,000 events were acquired. Gating strategy: cells were first gated on FSC-A vs. SSC-A to exclude debris, followed by FSC-A vs. FSC-W to select single cells. Compensation was performed using single-stained controls. Data were analyzed using FlowJo software (v10.8, TreeStar, Ashland, OR, USA).

### Enzyme-linked immunosorbent assays (ELISA)

We measured TNF-α, IL-1β and IL-6 in rat serum or cell supernatants using respective ELISA kits (Nanjing Jiancheng Bioengineering Institute, China) as described by the manufacturer. Staining intensity was determined as absorbance at 450 nm.

### Quantitative reverse transcription polymerase chain reaction (qRT-PCR)

Total RNA was isolated from collected tissues or cells using the TRIzol reagent (manufacturer name, city, country) as described by the manufacturer. Real-time quantitative reverse transcription PCR (qRT-PCR) proceeded using SYBR Green Master Mix (Roche Holdings AG, Basel, Switzerland) on a 7500 Fast Real-Time PCR System (Applied Biosystems, Waltham, MA, USA) and the primers shown in Table 1. U6 was the endogenous control. Relative gene expression was calculated using 2^−ΔΔCt^.

**TABLE 1:** Primers used for quantitative RT-PCR.

Primers (5′- 3′)	Sequence
GZMB forward	GA GCA​AGG​AGG​AAA​CAA​CAG​C
GZMB reverse	TGA​TCT​CCC​CTG​CAT​CTG​CC
U6 forward	GCT​TCG​GCA​GCA​CAT​ATA​CTA​AAA​T
U6 reverse	CGC​TTC​ACG​AAT​TTG​CGT​GTC​AT

### Western blotting

Proteins from tissues or cells were extracted and centrifuged at 4 °C and 12,000 rpm for 20 min, then the protein content was determined in the supernatant using Micro BCA Protein Assay Kits (Pierce Biotechnology Inc., Rockford, IL, USA). Proteins (40 µg) were transferred to PVDF membranes in transfer buffer and resolved SDS-PAGE. The separated proteins were transferred to polyvinylidene fluoride membranes. Nonspecific binding was blocked in Tris buffered saline containing 5% Tween 20% and 5% skim milk at room temperature for 1 h. The membranes were incubated overnight at 4 °C with the internal control 1:1,000-diluted mouse GAPDH (ab8245; Abcam, Cambridge, UK) and the primary antibodies to detect the GZMB, matrix metalloproteins (MMPs)-2 and 9, phosphorylated (p-AKT), AKT, p-PI3K, PI3K, p-mTOR and mTOR. The membranes were then incubated with secondary 1:5,000-diluted anti-mouse IgG antibody coupled with HRP (ab6728; Abcam). Target proteins were detected using EZ-ECL chemiluminescence detection kits (Pierce Biotechnology Inc.) as described by the manufacturer and the results were analyzed using ImageJ software (U. S. National Institutes of Health, Bethesda, Maryland, USA). Band intensities were quantified using ImageJ software (v1.53, NIH, Bethesda, MD, USA). For each membrane, background subtraction was performed using the “rolling ball” method (radius = 50 pixels). For phosphorylated proteins (p-PI3K, p-AKT, p-mTOR), the signal intensity was first normalized to the corresponding total protein (PI3K, AKT, mTOR) from the same membrane. Total protein levels were then normalized to GAPDH as a loading control. All quantifications were performed from three independent biological replicates (n = 3 separate experiments for cell studies; n = 3 tissue lysates per group for animal studies). Representative blots from one experiment are shown in the figures.

### Immunohistochemistry

The sections were washed with phosphate-buffered saline (PBS) and incubated overnight at 4 °C with an anti-GZMB antibody (1:100; ab4059; Abcam; China). Subsequently, the sections were washed three times in PBS for 5 min and incubated with biotinylated goat anti-rabbit immunoglobulin G (IgG) (1:1000; ab150077; Abcam, China) at room temperature for 20 min. The sections were washed with PBS, and an avidin-horseradish peroxidase complex was added (SABC Kit; Bost, Wuhan, China). The 3,3-diaminobenzidine was used as a chromogen. Positive cells were visualized under a light microscope.

### Statistical analysis

Data were statistically analyzed using GraphPad Prism 8.0 (GraphPad Software Inc.). Results are presented as means ± standard deviation. For comparisons involving more than two groups, one-way analysis of variance (ANOVA) was performed, followed by Tukey’s *post hoc* test for multiple comparisons. All P-values reported for multi-group comparisons were adjusted accordingly. A P-value <0.05 was considered statistically significant.

## Results

### Expression of GZMB was elevated in MH7A cells

**FIGURE 1:**
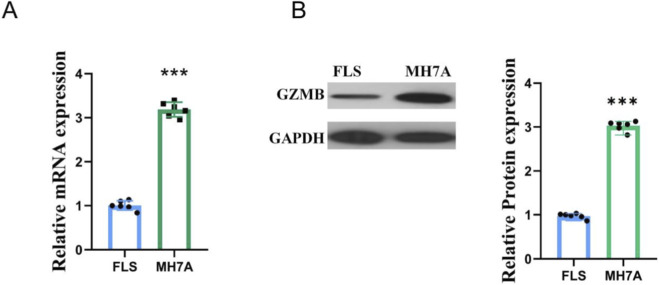
Expression of GZMB in MH7A cells. **(A)** qRT-PCR showing messenger RNA expression of GZMB in MH7A cells. **(B)** Western blot showing protein expression of GZMB in MH7A cells. n = 6. Data are expressed as the mean ± SD. For two-group comparisons, Student’s t-test was applied. Results indicate a significant decrease in comparison with the FLS. ***P < 0.001.

First, we investigated GZMB expression in MH7A cells. Whether at the mRNA level or the protein level, we found significantly more GZMB in MH7A cells than in FLS (Figure 1).

### Knockdown of GZMB in MH7A cells

**FIGURE 2:**
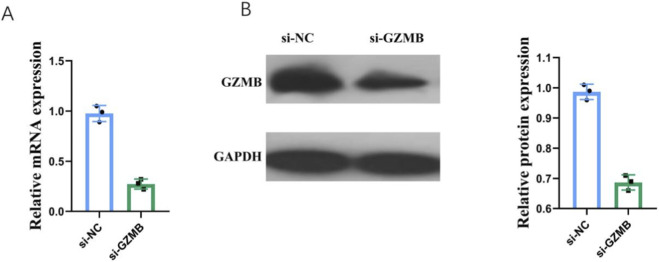
**(A)** qRT-PCR showing relative mRNA expression of GZMB in MH7A cells transfected with si-GZMB. **(B)** Western blot showing relative protein expression of GZMB in MH7A cells transfected with si-GZMB. si-NC: MH7A cells treated with si-NC; si-GZMB: MH7A cells treated with si-GZMB. n = 3. Data are expressed as the mean ± SD. For two-group comparisons, Student’s t-test was applied. Results indicate a significant decrease in comparison with the si-NC. ***P < 0.001.

In addition, Figures 2A,B showed that GZMB was effectively knocked down in MH7A cells after transfection with si-GZMB.

### Knockdown of GZMB inhibits proliferation, inflammatory responses, migration and invasion in MH7A cells *in vitro*

**FIGURE 3:**
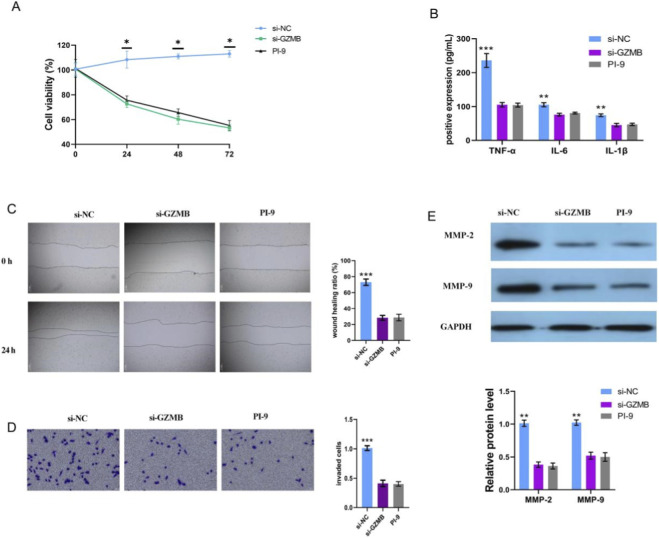
Downregulated GZMB expression inhibited proliferation and inflammatory responses of MH7A cells. **(A)** Proliferation of MH7A assessed using Cell Counting Kit-8 (CCK-8) at 0, 24, 48, and 72 h cells after GZMB downregulation. **(B)** Changes in cytokines determined by ELISA in MH7A cells incubated with si-NC or with si-GZMB. **(C,D)** Inhibited migration and invasion of MH7A cells transfected with si-GZMB. **(E)** Western blot showing that GZMB knockdown modestly but significantly MMP-2 and MMP-9 protein levels. si-NC: MH7A cells treated with si-NC; si-GZMB: MH7A cells treated with si-GZMB. n = 3. Data are expressed as the mean ± SD. For comparisons involving multiple groups, one-way ANOVA with Tukey’s *post hoc* test was used. Results indicate a significant decrease in comparison with the si-NC. *P < 0.05; **P < 0.01; ***P < 0.001.

Subsequently, we detected the downregulation of GZMB at the cellular level. The proliferation of MH7A cells incubated with GZMB knockdown decreased according to the CCK-8 results (Figure 3A). Additionally, the results showed that TNF-α, IL-1β, and IL-6 expression in MH7A cells were all decreased, when incubated with GZMB knockdown compared with control group (Figure 3B). Migration experiments showed that cell mobility was lower in MH7A cells with GZMB knockdown compared with the si-NC group, indicating that GZMB knockdown inhibited cell migration (Figure 3C). Transwell assays revealed that the number of invasive cells was significantly reduced in the in the group with GZMB knockdown (Figure 3D). Moreover, the levels of MMP-2 and MMP-9 were modestly but significantly decreased in the group with GZMB knockdown compared with si-NC (Figure 3E).

### Knockdown of GZMB is associated with MH7A cell apoptosis

**FIGURE 4:**
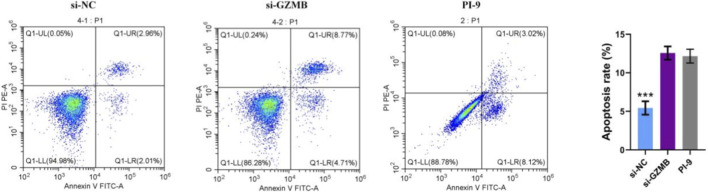
Effects of GZMB downregulation on cell apoptosis determined using flow cytometry. si-NC: MH7A cells treated with si-NC; si-GZMB: MH7A cells treated with si-GZMB. n = 3. Data are expressed as the mean ± SD. For comparisons involving multiple groups, one-way ANOVA with Tukey’s *post hoc* test was used. Results indicate a significant decrease in comparison with the si-NC. ***P < 0.001.

Flow cytometry analysis performed 48 h post-transfection revealed that the percentage of apoptotic cells was significantly increased in the si-GZMB group compared with the si-NC control group. Similarly, treatment with the GZMB inhibitor PI-9 also increased apoptosis compared with the control group, consistent with the knockdown results (Figure 4). These findings indicate that inhibition of GZMB, is associated with increased apoptosis of MH7A cells.

### Knockdown of GZMB alleviates injury in RA rats

**FIGURE 5:**
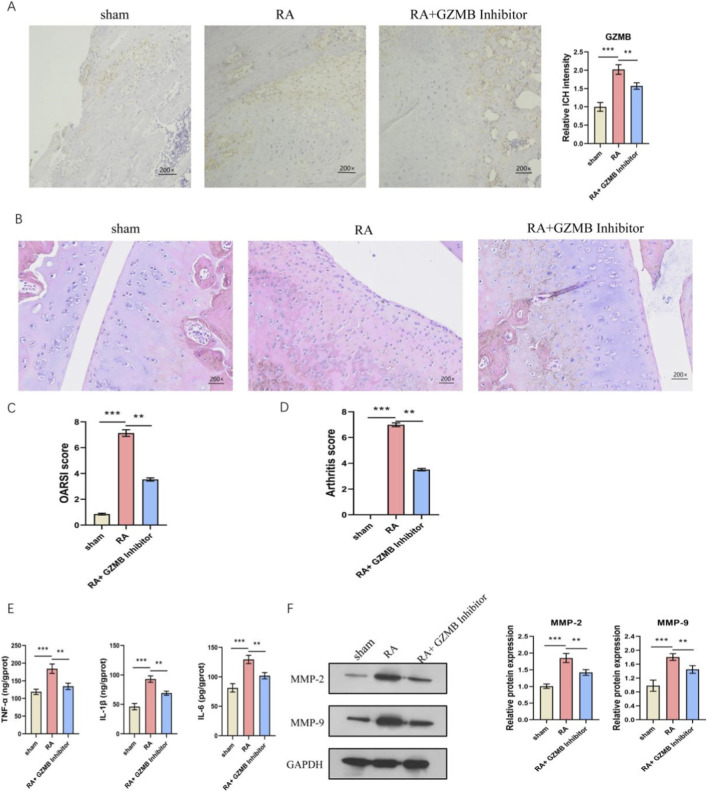
Knockdown of GZMB alleviates injury in RA rats. **(A)** Immunohistochemistry staining reflected the expression of GZMB. **(B)** Hematoxylin and eosin staining of each group. **(C)** The changes of arthritis score in each group were estimated. **(D)** The OARSI scores were estimated in each group. **(E)** ELISA was used to determine changes of serum cytokines in each group. **(F)** Western blot was used to determine changes of MMP-2 and MMP-9 in each group. n = 6. Data are expressed as the mean ± SD. For comparisons involving multiple groups, one-way ANOVA with Tukey’s *post hoc* test was used. Results indicate a significant decrease in comparison with the arthritis.**P < 0.01; ***P < 0.001.

To verify the effects of GZMB *in vivo*, we established a rat model of RA. Immunohistochemical results showed that the expression of GZMB was significantly increased in the synovial tissue of the joints of RA rats. However, these levels decreased after treatment with the GZMB inhibitor (Figure 5A). Additionally, the HE staining results showed that in the sham group, articular cartilage and articular capsule structure were normal, articular cartilage surface was smooth, synovial tissue morphology was regular, and no clear abnormalities were observed. In the RA group, some articular cartilage surfaces were rough, synovial tissue hyperplasia was evident with many infiltrating inflammatory cells, and pannus formation damaged the articular surface. However, compared to the RA group, the articular cartilage surface of rats in the GZMB inhibitor group was smooth, and synovial tissue hyperplasia and inflammatory cell infiltration were reduced (Figure 5B). The arthritis and OARSI scoring results indicated that GZMB inhibitor significantly reduced in RA rats (Figures 5C,D). Moreover, the TNF-α, IL-1β, and IL-6 levels in the peripheral blood of the RA rats were elevated, whereas GZMB inhibitor treatment partially reduced the expression levels of these cytokines (Figure 5E). As shown in Figure 5F, the protein expression of MMP-2 and MMP-9 was abnormal in the RA group. However, these effects were reversed upon treatment with a GZMB inhibitor.

### Knockdown of GZMB reduced expression of PI3K/AKT/mTOR signaling pathway

**FIGURE 6:**
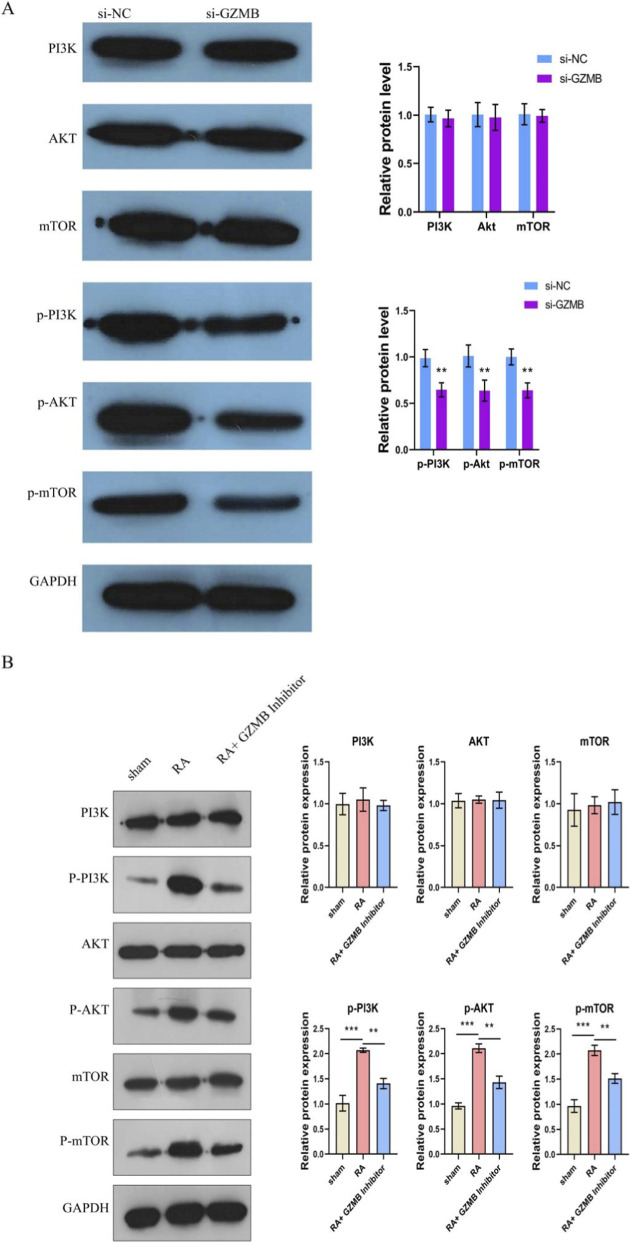
Downregulation of GZMB-induced inhibition of the PI3K/AKT/mTOR pathway. **(A)** Western blots of total protein and phosphorylated protein levels of PI3K/AKT/mTOR pathway in cells incubated with si-GZMB. **(B)** Western blot analysis showing PI3K/AKT/mTOR pathway protein expression levels in RA model rats with GZMB inhibitor treatment. n = 6. Data are expressed as the mean ± SD. For two-group comparisons, Student’s t-test was applied. For comparisons involving multiple groups, one-way ANOVA with Tukey’s *post hoc* test was used. **P < 0.01; ***P < 0.001.

We evaluated activation of the PI3K/AKT/mTOR pathway to uncover possible molecular mechanisms underlying the downregulation of GZMB-mediated inhibition of RA. As shown in Figure 6A, knockdown of GZMB in MH7A cells significantly reduced the p-PI3K/PI3K, p-AKT/AKT, and p-mTOR/mTOR ratios compared with the si-NC control group. In contrast, total protein levels of PI3K, AKT, and mTOR did not differ significantly between the si-NC and si-GZMB groups (Figure 6A), indicating that GZMB knockdown specifically suppresses the activation rather than the expression of this signaling pathway. Consistent with the *in vitro* findings, RA rats treated with the GZMB inhibitor showed significantly lower p-PI3K/PI-3K, p-AKT/AKT, and p-mTOR/mTOR ratios in synovial tissue compared with untreated RA rats (Figure 6B). No significant differences in total PI3K, AKT, or mTOR protein levels were observed among the sham, RA, and GZMB inhibitor groups (Figure 6B). Taken together, these results demonstrate that GZMB knockdown suppresses activation of the PI3K/AKT/mTOR signaling pathway both *in vitro* and *in vivo*.

## Discussion

Rheumatoid arthritis is a chronic systemic disease of unknown etiology. It is mainly associated with the destruction of articular cartilage and surrounding tissues, characterized by synovial hyperplasia and inflammatory synovitis (Huang et al., 2021). Granzyme B might be associated with the development of RA (Zhang et al., 2023). The MH7A cells are RA-derived immortalized synoviocyte line, transformed from fibroblast-like synovial cells of RA, possessing the pathological characteristics of RA. It is used in the study of the pathogenesis and treatment cell model of RA (Ba et al., 2021; Li et al., 2022). We found upregulated GZMB expression in MH7A cells, and that GZMB knockdown reduced the intracellular expression of inflammatory factors and inhibit cell proliferation. These results suggested that GZMB knockdown functions by inhibiting synovial hyperplasia and the inflammatory response to RA. Furthermore, abnormal cell cycles and insufficient apoptosis are also causes of RA. We found that GZMB knockdown can reduce the viability of MH7A cells and increase the apoptosis rate of MH7A cells. Therefore, GZMB inhibits the progression of RA at the cellular level.

The results in the present study demonstrate that knockdown of GZMB in MH7A cells suppresses their proliferation, migration, invasion, and inflammatory cytokine production. At first glance, this appears to contradict reports suggesting that GZMB may be protective against joint destruction (Bao et al., 2018). However, these seemingly opposing findings can be reconciled by considering the compartmentalization of GZMB action. Intracellular GZMB, delivered by CTLs or NK cells, induces apoptosis of target cells, a mechanism that could potentially eliminate pathogenic autoreactive lymphocytes or hyperplastic FLS. In contrast, extracellular GZMB, which can be released from degranulating immune cells or secreted by activated FLS, degrades cartilage matrix components and activates pro-inflammatory mediators. The present study targeted endogenous GZMB within FLS, likely reducing both intracellular stores available for secretion and extracellular activity in the synovial microenvironment. This may explain why GZMB knockdown was beneficial in our experimental system.

Inflammation stimulates FLS to undergo tumorlike cell transformation in the synovial lining during RA (Debreova et al., 2022). Thereafter, FLS proliferation, migration and invasion are significantly enhanced, and secreted pro-inflammatory factors and chemokines aggravate bone and cartilage erosion and eventually lead to irreversible joint breakage (Bartok and Firestein, 2010). Therefore, inhibiting the proliferation, migration and invasion of FLS has become one of the strategies to intervene in RA. We found that GZMB inhibited the migration and invasion of MH7A cells. Among the MMP family, MMP-2 and MMP-9 can damage the extracellular matrix of cartilage tissue by degrading collagen types IV and V, gelatin, and elastin, which leads to the destruction of bones and joints. These MMPs are markers of cell migration and invasion that mediate the abnormal migration and invasion of tumor cells (Li et al., 2019). We found that knockdown of GZMB reduces MMP-2/9 and attenuates migration/invasion, suggesting that endogenous GZMB promotes malignant-like RA-FLS behavior.

We constructed a rat model of collagen-induced RA to evaluate the therapeutic potential of GZMB inhibitors in RA *in vivo*. We observed that the articular cartilage surface of RA rats treated with the GZMB inhibitor recovered, and the degree of synovial tissue hyperplasia and inflammatory cell infiltration was reduced, indicating that the GZMB inhibitor promoted the decrease of the disease. RA is a chronic autoimmune disease, and inflammation plays a key role in its development (Radu and Bungau, 2021). In the context of an imbalance in immune system regulation, autoantigens are misrecognized, thereby activating the immune response. The activation of T and B lymphocytes leads to an increase in the production of pro-inflammatory factors, which further activate synovial cells, enhance the permeability of synovial blood vessels, and attract more immune cells into the joint cavity, resulting in synovitis (Lin et al., 2020). Inflammatory cells accumulate in the joints and release enzymes and free radicals that damage cartilage and bone, thus contributing to the development of the disease. Sustained immune activation in the context of slow inflammation encourages synovial cells to gradually form aggressive synovial tissue similar to tumors (Nerurkar et al., 2019). Abnormally elevated expression of IL-1β and IL-6 leads to activation of the downstream inflammatory cascade (Kondo et al., 2021). In this study, it was observed that the expressions of pro-inflammatory factors IL-1β and IL-6 in the serum of RA mice treated with GZMB inhibitor were significantly inhibited, suggesting that GZMB inhibitor may participate in the treatment of RA by inhibiting the expression of inflammatory mediators and inhibiting the inflammatory response at the joint.

The activated PI3K/AKT signaling pathway causes synovial inflammation in joints and erodes bone and cartilage by promoting the tumor-like biological behavior of RA-FLS (Cheng et al., 2022). Therefore, blocking the PI3K/AKT pathway can alleviate RA development. Here, downregulated GZMB reduced p-PI3K and p-AKT expression in MH7A cells and RA rats, suggesting that it also inhibited the migration and invasion of synoviocytes by regulating the PI3K/AKT pathway, thereby alleviating RA. This is the first report indicating that the downregulation of GZMB in RA may be associated with the PI3K/AKT pathway. We will further explore the relationship between them in our future research.

This study had some limitations. We investigated the effects of GZMB knockdown on the regulation of PI3K/AKT expression. However, further verification using pathway activators is needed to confirm the regulatory effect on expression. Moreover, whether GZMB actually alleviates RA by activating the PI3K/AKT pathway awaits further exploration. In addition, subsequent clinical trials are needed to further demonstrate the potential of low-expression GZMB for the treatment of RA. Furthermore, the present study did not directly measure extracellular GZMB activity or distinguish between the intracellular and extracellular pools. Future studies using GZMB protease inhibitors or activity-based probes are needed to determine which compartment drives the pathogenic effects observed here.

## Conclusion

We found that GZMB knockdown inhibits the proliferation, migration, invasiveness and inflammatory responses of MH7A cells, and promotes cell apoptosis. Additionally, the downregulation of GZMB alleviated histopathological changes and inflammatory responses in RA rats. Furthermore, downregulated GZMB to regulate the PI3K/AKT pathway and inhibit the expression of MMP-2 and MMP-9 proteins are possible mechanisms of these actions. Our findings provide a novel therapeutic target and strategy with which to treat RA.

## Data Availability

The original contributions presented in the study are included in the article/supplementary material, further inquiries can be directed to the corresponding author.
